# Young people, mental health practitioners and researchers co-produce a Transition Preparation Programme to improve outcomes and experience for young people leaving Child and Adolescent Mental Health Services (CAMHS)

**DOI:** 10.1186/s12913-017-2221-4

**Published:** 2017-04-20

**Authors:** Valerie Dunn

**Affiliations:** Department of Psychiatry NIHR CLAHRC East of England/University of Cambridge, 18b Trumpington Road, Cambridge, CB2 8AH UK

**Keywords:** Mental health services, Young people, CAMHS transition, Mental health, Participatory research, Collaboration, Transition preparation

## Abstract

**Background:**

In the UK young people attending child and adolescent mental health services (CAMHS) are required to move on, either through discharge or referral to an adult service, at age 17/18, a period of increased risk for onset of mental health problems and other complex psychosocial and physical changes. CAMHS transitions are often poorly managed with negative outcomes for young people. Better preparation may improve outcomes and experience. This study aimed to co-produce, with young people who had transitioned or were facing transition from CAMHS, a CAMHS Transition Preparation Programme (TPP), deliverable in routine NHS settings.

**Methods:**

Eighteen young people, aged 17–22, from three UK National Health Service (NHS) mental health foundation trusts participated in creative, participatory research workshops. Seven parents completed short questionnaires. Thirty clinical staff from two trusts took part in workshops to ensure deliverability of young people’s ideas. Young people were offered co-research opportunities.

**Results:**

Most young people felt anxious, fearful and uncertain on leaving CAMHS and perceived mental health services as uncaring. Participants outlined transition procedures and drafted a range of preparation activities, centred around dedicated Transition Peer Support and a transition booklet, which should be offered to **all** CAMHS leavers, irrespective of discharge or transfer to an adult service. Preparation should aim to build confidence to help young people take responsibility for themselves and flourish in the adult world: coping or getting through it was not enough. Some clinicians also felt anxious at transition and recognised the potential impact on young people of poor communication and lack of understanding between services. Parents would appreciate help to support their offspring during the transition period. Clinicians cited lack of funding and inflexible NHS procedures and policies as potential barriers to the implementation of young people’s ideas. Nine young people took up co-research opportunities.

**Conclusions:**

Mental health services underestimate the anxiety of CAMHS leavers. Young people have clear ideas about the preparation they require to leave CAMHS with the confidence to take responsibility for their own health care. Close collaboration of NHS staff and researchers facilitates the implementation of research findings.

## Background

Although a few pioneering UK regions have transformed mental health services to offer provision to young people up to the age of 25, most require young people attending Child and Adolescent Mental Health Services (CAMHS) to transition to an adult service or discharge at age 17/18. Consensus is growing that this age-based cut-off, albeit convenient for service providers, is not in the best interests of young people [1]. It occurs during a period of increased risk for onset of mental health difficulties and young people are negotiating complex physical and psychosocial changes and demands. The system offers little flexibility to account for individual differences in maturity, readiness, functioning, wellbeing or life context.

The TRACK study, the UK’s pre-eminent investigation into transitions from CAMHS to adult services, found poor continuity of care, preparation and planning, resulting in adverse outcomes and negative service-user experience. TRACK identified policy/practice gaps, a clash of cultures between CAMHS and adult mental health services (AMHS), with poor understanding and communication between them. Many young people leave CAMHS in poor mental health, feeling unprepared for the move [2–7].

Models to assess preparedness and readiness to transition have been piloted with young people and adults in physical health [8–11], severe mental health needs [12] and inpatient settings [13], but are rare in community CAMHS. To our knowledge there are no standard, routine assessments of readiness for transition from CAMHS and robust transitional care models have yet to be developed or evaluated [4].

Policy, research and guidance agree on the cornerstones of good transition practice: joint working, thorough transfer of information, continuity of care, support and appropriate parental involvement during a gradual, tailored, flexible processes and preparation [2, 4, 14–18]. Details are scant, however, as to what preparation should involve, aim to achieve or what young people would find helpful and engaging.

The participation of service users in the design of the services which affect them is embedded within National Health Service (NHS) England policy and practice, and is core to the implementation of NHS England’s Five Year Forward View for Mental Health [18]. Participation is also a requirement in health services research. The aim is to research ‘with’ rather than ‘on’ or ‘to’ people [19], in recognition of the equal contributions of researchers and participants. Participatory methods have been recommended to explore transition support models with young people [14].

Participatory research (PR) approaches, rooted in community development, are democratic, collaborative and aimed to redress the researcher/subject power imbalance [20]. Under the PR umbrella sits a range of innovative, creative and arts-based techniques which do not rely exclusively on verbal or written competencies. These less conventional approaches recognise that ‘some knowings cannot be conveyed through language’ [21] and may be particularly appropriate when working with more vulnerable groups to facilitate meaningful exploration of complex or sensitive material [22]. Participants have time and space to think creatively, reflect, discuss and generate ideas, in contrast to traditional research approaches which usually require on-the-spot responses.

The aim of this 12-month study was to co-devise, with service users and practitioners, a CAMHS Transition Preparation Programme (TPP) deliverable in routine NHS settings. The study was designed to maximise collaborative working. The ‘bottom-up’ approach set out to empower young people to generate original material rather than simply comment on existing policy, practice and guidance. This paper describes the process of co-producing the programme with young people, and the features and components of the proposed programme.

## Methods

In order to facilitate the co-production of the programme, a series of workshops was delivered to young people with CAMHS experience and then to clinical staff from child/adolescent and adult directorates in three NHS mental health trusts. Workshops were delivered in stages to allow time for data synthesis and review. At the design stage, the NIHR Clinical Research Network: Mental Health’s Young People’s Advisory Group and participation networks in two participating trusts were consulted about the study design and research topic.

Workshops were co-designed by Tom Mellor, workshop designer/facilitator and the author, who had previously collaborated on projects with young people in care. Tom, who facilitated the workshops, assisted by the author, brought considerable experience working creatively with groups of young people in youth offending and school settings and with adults in custody. The author designed the study, building on a previous CLAHRC study on transition [2], managed the project, co-designed and co-facilitated workshops, produced consensus documents and led dissemination.

The study was independently evaluated by Caroline Lee, evaluation researcher at the University of Cambridge, Department of Public Health. Caroline was not involved in the planning, design or delivery of the work. She evaluated the work through observation, short evaluation questionnaires, focus groups and interviews with young people, NHS participation coordinators (PCs), the workshop facilitator and the author [23].

### Setting and participants

Three NHS Mental Health Foundation Trusts, Norfolk and Suffolk, Cambridgeshire and Peterborough and Hertfordshire University Partnership (NSFT, HPFT, CPFT), were invited, and agreed, to participate. Each was a partner in the NIHR CLHARC East of England, a coalition of universities, NHS and social care organisations, who collaborate on applied health research [24]. Although, in 2013 NSFT transformed their mental health provision to offer a youth service for 14–25 year olds, the participants from this trust had transitioned under the traditional system, leaving CAMHS at 17.

Each trust supported a Young People’s Participation Network to involve CAMHS-experienced young people in a range of activities to improve and shape the design delivery of their services. Networks were run by designated PCs who were integral to this research. Their expertise, knowledge, insight and experience was invaluable in the workshops, as was the support, both practical (organising transport, venues and dates) and emotional, they offered young people throughout the study. PCs also handled recruitment in their respective trusts.

Eligible young people were aged 16–22, had transitioned, or were approaching transition, from CAMHS and were involved in participation networks. In two trusts the networks were well established and here the PCs introduced the study and distributed flyers at routine youth meetings and activities. In the third trust the participation network had been operational for less than 12 months. A core group with a programme of routine meetings had not been established but the PC kept an electronic list of volunteers who she contacted for specific activities. Here, the PC emailed and telephoned eligible young people on her list and circulated flyers electronically. She also displayed flyers in the CAMHS waiting room. In all trusts, young people expressing an interest met with their PC and the author for full details and information packs and were encouraged to discuss participation with a trusted adult before providing written consent. All those who expressed an initial interest were invited to pass on short questionnaires to parents asking for their views on transition preparation. Consenting young people were asked to complete short demographic questionnaires which included short free-text boxes for opinions about transition which informed the workshop designer and author in the design of the workshops.

Clinical staff were invited to participate in workshops in order to gather their views on potential barriers to implementation of the programme and the deliverability of the young people’s recommendations. The author contacted service managers in adult and child/adolescent directorates to recruit clinical staff. Following initial email contact, the author was invited to present the study at routine team and management meetings. In one trust the workshop ran in place of a routine team meeting and in the other a late afternoon slot was chosen for convenience. Clinician sessions did not run in NSFT due to the major service transformation in the trust.

Young people were offered opportunities to be involved as co-researchers: co-designing a conference poster for the Royal College of Physicians Conference on developmentally appropriate care at transition, disseminating findings both within trusts and externally, co-planning and co-hosting the clinician workshops, co-authoring a journal article and reviewing research literature. Bespoke literature search training was provided by Dr Isla Kuhn, Medical Librarian at the University of Cambridge Clinical School library.

### Workshops, data synthesis and consensus

**Fig. 1:**
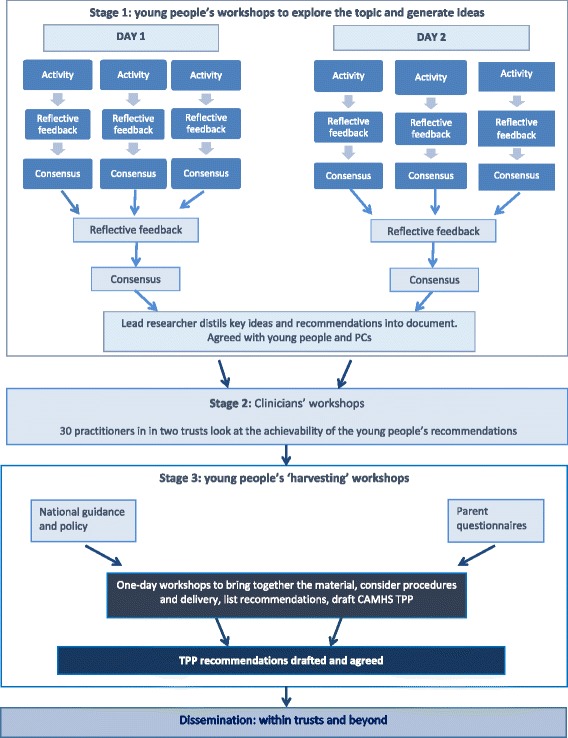
Study design

Creative, participatory workshops were delivered at three stages, illustrated in Fig. 1. Two months separated the first and second stages with the third stage taking place one month later. Workshops were designed and run by the workshop facilitator and author, supported by PCs and observed by the evaluation researcher. The ‘data’ generated by young people took the form of drawings, posters, lists, maps, characters, leaflets and timelines. The material required careful synthesis rather than complex analysis.

#### Stage 1

Two-day workshops were held in each Trust during school holidays. The aim was to inspire young people to think creatively about the issues, explore and share experiences, provoke discussion and generate ideas and recommendations for transition preparation. Confidentiality agreements, routinely used in all trust participation activities, were agreed at the outset of each workshop, displayed clearly on the wall and revisited at each new session. Each day a series of balanced activities was designed to maintain momentum and energy, to build confidence and positive, trusting group dynamics. Activities, including games, body-mapping, character creation, role plays, timelines, leaflet and poster design, were drawn and adapted from teaching, drama and group work practice and our work with young people in care [25, 26]. Activities were designed to introduce topics in novel ways and each was followed by a scaffolded discussion, led by the workshop facilitator, to build meaning and identify common themes and experiences. Table 1 describes some core activities.

**Table 1:** Brief descriptions of core workshop activities

Activity	Aim/s	Practice
Transition mapping: what can we learn from previous transitions?	To learn from previous transitions (default = primary-secondary school).	In pairs/small groups young people identify other transitions in life: what preparation was offered? What worked/didn’t work, why? How did you feel? What was left out? On large sheets of paper young people map the processes in their chosen format (mind-map, diagram, list, poster). Feedback to whole group, sheets posted on walls. **SD:** what was useful/not useful and why, what translates to CAMHS transition.
Body mapping/character creation. (A life-sized outline of a person produced by drawing around a volunteer, given name and full back story based on experience.)	To co-create a character to represent the experiences of the group; to provide a means for discussion of personal experiences from a safe distance.	In small groups or pairs young people created characters facing transition, which grew to reflect the joint experiences of the group. A variety of coloured post-it notes denoted particular themes, e.g. questions, decisions, worries, life events, mental health problems. Characters represent pooled group experiences. **SD:** sub-groups/pairs fed back to the main group describing the characters’ lives and experiences. and characters were referred to regularly throughout and ‘imagined’ in specific situations under discussion. Clinicians also created characters.
Socks game: group juggling	Metaphor to illustrate the multiple demands of teenage life: prioritising, juggling, coping, dealing with conflicting demands, feelings, react to surprises.	Led by the workshop facilitator, ‘socks’ is a whole group game involving a repeating circuit of throwing and catching of increasing numbers of balls of socks (could be anything soft), until a crescendo of near-chaos is reached, when the facilitator gradually slows the pace and restores calm. **SD**: built from initial reactions and responses ‘*how did it feel when you had lots of socks coming at you at once*?’ into a wider discussion about real-life juggling of demands/pressures/events (as represented by the socks) of adolescence, responsibilities and coping strategies. Sometimes though, by request, the game was played purely for fun. Clinicians also played ‘socks’.
Anti-model: produce poster/leaflet for ‘worst’ mental health service	To stimulate thought and generate discussion on what makes a gold standard mental health service.	Small groups/pairs designed a poster/leaflet advertising the ‘worst’ mental health service. They played with exaggeration, cartoon, jokes, puns. FB to whole group, display work. **SD:** by discussing the anti-model, aspects of a gold standard model emerge.
Lego communication game	To stimulate discussion about talking about difficult things: finding the right words, responsibilities of listener and speaker, assumptions, reading between the lines.	In pairs young people sit back-to-back. Person A is given an abstract lego model and B a large pack of lego bricks including the pieces necessary to replicate the model. A instructs B who tries to reproduce the model. Swap. **SD:** finding the right words, accuracy, difficulties, responsibilities, broadened to the difficulties expressing feelings/emotions, talking to a therapist, parent, saying what you mean, asking for help.

*FB* feedback, *SD* scaffolded discussion

Through a process of ongoing consensus, young people were involved in distilling the material, identifying themes and action points to take forward: after each activity, each workshop day and at the end of each workshop stage, the workshop facilitator and researcher led reflective feedback discussions by the end of which, the core points to take forward had been agreed by the group. After the three stage 1 workshops, the author drew together the key points in a document which was circulated to PCs and onwards to participants, in each trust for comment and agreement and to ensure the views of each group were fairly represented.

#### Stage 2

*S*tage 2 workshops ran in two trusts for clinical staff from child/adolescent and adult mental health services. The aim was to examine practitioner perceptions of barriers and enablers to delivering best practice in transition, to discuss young people’s ideas and advise on the achievability of the young people’s ideas. The key discussion points were those agreed at stage 1. Workshops were two to three hours and facilitated by the workshop facilitator, co-hosted with young people who were keen that the format be active and participatory to parallel the young people’s sessions. After an introduction, in small groups clinicians took part in character creation exercises to reflect a CAMHS practitioner’s views and experiences of CAMHS transition. On each character, clinicians drew and wrote their questions, concerns, experiences and barriers to successful CAMHS transition. Small groups fed back to the main group and themes were discussed and noted by the author. Some young people chose to make their own notes. The young people’s ideas which had been generated at stage 1, were presented to clinicians on A5 sheets with free-text boxes for their views on achievability. After the workshop, in preparation for stage 3, these were collated into a summary document by the author.

#### Stage 3

In these one-day ‘harvesting’ workshops, participants distilled the material from previous stages, considered procedures, parent questionnaire responses, generated core recommendations and drafted a Transition Preparation Programme. The author introduced key points from relevant national policy and guidance for consideration and young people discussed the youth service model which offers support up to age 25. Finally, the lead researcher wrote a detailed project report which was emailed to staff and young people via PCs in each trust. No amendments or comments were received.

## Results

Seventeen young people (12 females, four males, one gender fluid) participated in the study: four in NHS trust one (Tr1), nine in trust two (Tr2), four in trust three (Tr3). Participants in Tr3 participated in stage 1 workshops only due to conflicting commitments, including major fundraising events and attendance at an international conference. Table 2 shows participation in more detail.

**Table 2:** Participation in each stage of the study

Total participants:		
-Young people	17	Tr1 = 4; Tr2 = 9; Tr3 = 4
-Clinicians attending workshops	30	Tr1 = 22; Tr2 = 8
-Completed parent questionnaires	7	Tr1 = 3; Tr2 = 1; Tr3 = 3
Young people’s participation:		
-Stage 1 workshops	13	Tr1 = 4; Tr2 = 5; Tr3 = 4
-Stage 2 clinician workshops	6	Tr1 = 1; Tr2 = 5
-Stage 3 ‘harvesting’ workshops	8	Tr1 = 2; Tr2 = 6
Young people taking up co-researcher roles:	9	Tr1 = 2; Tr2 = 7
Of which:		
-Literature search training	5	Tr2
-Co-designed/hosted clinician workshops	6	Tr1 = 3; Tr2 = 3
-Co-presented to steering group	3	Tr2
-Transition review panels	4	Tr1 = 2; Tr2 = 2
-Co-designed conference poster	1	Tr1
-Co-presented research training workshop	2	Tr1
-Co-presented to Trust Board Meeting	2	Tr1
-Co-authored paper for publication	1	Tr2

All participants were recruited from existing participation networks. The waiting room posters in Tr1, where the network was less well established, did not attract any participants. In Tr1 the same individuals participated at each stage but only two of the original four remained involved throughout. Of the nine participants in Tr2: three participated at all stages, and, of those, one took part in all co-researcher opportunities; four young people who were unavailable for the stage 1 workshop, joined the study when they were free, participating in co-researcher activities and two joined the stage 3 workshop; four of the five who took part in the stage 1 workshop also took part in the stage 3 harvesting session. In both trusts, participation in various activities was dictated by lack of availability and ill health rather than lack of interest.

Eleven young people completed demographic questionnaires. The median age was 18.75 years (range 16.4–22.6). Seven participants were students and four were not in education, employment or training (NEET). Participants had attended CAMHS for 12–84 months, five had been discharged to primary/self-care, four had transferred to AMHS, one was currently attending CAMHS and one didn’t say. Nine gave reasons for attending CAMHS: depression/anxiety (*n* = 3); psychotic illness (*n* = 1); depression/anxiety/ self-harm (*n* = 1); autism/anxiety/depression/pain condition (*n* = 1); young carer/anger/family (*n* = 1); anorexia nervosa/depression/anxiety/auditory/visual hallucinations (*n* = 1); clinical depression/Aspergers/self-harm (*n* = 1).

Nine young people took up co-researcher opportunities. Bespoke literature search training was provided by at the University of Cambridge Clinical School library to five young people. Although the young people were enthusiastic during the training, none carried out subsequent literature reviews. The duration of the study was only 12 months which proved insufficient time for participants to receive training or to carry out the planned thematic analysis of parent questionnaires. This is discussed in limitations. In two trusts the author, PCs and young people fed research findings into ongoing CAMHS transition review panels.

### Young people’s experiences of mental health services and CAMHS transition

A number of related issues emerged from the workshops which informed the design and content of the TPP.

#### Uncaring CAMHS

Most young people perceived CAMHS and AMHS as uncaring, prioritising provider convenience over the interests of young people. Administrative errors, long waiting times, frequent staff changes, lack of information, not being kept informed and poor or inappropriate (non-young-person friendly) facilities were taken as evidence of indifference and lack of care. Many felt they had not been listened or even believed, excluded from decisions and were uncomfortable when clinical staff discussed them ‘*behind closed doors’*. Those less confident or shy felt over-looked and struggled to find their voice in a complex system.

Two participants, both discharged to primary care, reported positive leaving experiences characterised by good clinician relationships, ample information and support: they had felt sufficiently well, confident and able to cope. One had received highly-valued support from a private life coach for over 12 months, funded by parents. Most young people, however, had left CAMHS feeling anxious, uncertain and fearful – about adult services, relapse, sources of support and their ability to cope. Many had felt ill-informed, uninvolved and ‘*abandoned*’ or ‘*chucked out’*. Transitions had been abrupt and treated separately from other life experiences.

Many clinicians also voiced anxiety about transition and were uncertain about referral criteria to adult services and alternative sources of support. Many felt guilty when transition didn’t go well, ‘*we sometimes feel like we’re abandoning them’*.

#### CAMHS/AMHS divide

All parties identified a CAMHS/AMHS divide: ‘*If we don’t understand each other, how can we help young people*?’ [clinician]. Clinicians recognised different service cultures: developmental vs diagnostic, nurturing and protective vs autonomous and taking personal responsibility with insufficient information, poor communication and lack of understanding of their respective cultures, practices, expectations and language. Many AMHS clinicians lacked confidence with young people. As clinician workshops progressed, discussions grew increasingly solution focused with talk of ‘*meeting in the middle*’. One AMHS clinician tentatively broached the possibility of a shift in approach more attuned to the particular needs of emerging adults. Similarly, a CAMHS clinician pondered ‘…*are we too nurturing, should be we encouraging young people to take more responsibility for themselves*?’ Joint CAMHS/AMHS training, with input from young people, was suggested as a positive way forward.

Many young people were also confused by what they saw as inconsistent and confusing differences in approaches between CAMHS and AMHS: ‘i*t’s the same thing, how can it be one thing here and something different there*?’ For example, one young person felt her self-harm had been well-supported at CAMHS and viewed as a sign of distress. On transfer to AMHS however, she felt it was simply recorded as a matter of personal choice. She had felt confused, angry and frustrated but lacked the confidence to speak up or ask questions.

### Barriers identified by clinicians

Thirty CAMHS and AMHS staff in two trusts - consultant psychiatrists, clinical psychologists, occupational therapists, single points of access staff and team managers - discussed the achievability of young people’s ideas and their own perceptions of barriers and facilitators to transition. Clinicians viewed most of the young people’s ideas favourably but with reservations ‘*… FUNDING will always be an obstacle!!’.* Inflexible NHS policies and structures and embedded cultures were also cited as barriers which require ‘*clear steer and buy-in from commissioners*’. Clinicians identified a policy/practice gap and called for more support to honour transition policy. Among suggestions were: routine joint CAMHS/AMHS team meetings to improve and maintain channels of communication and improve inter-directorate understanding; information about the quality of support offered by third sector organisations; training for AMHS practitioners on the particular needs of emerging adults; time and resources for improved, joint training; the introduction, **before** transfer to AMHS is mentioned to a young person, of routine conversations between referring CAMHS and accepting AMHS clinicians to ascertain the likelihood of acceptance by AMHS.

### The aims of the CAMHS Transition Preparation Programme

Young people decided the aims of transition preparation should be ambitious: coping or getting through it is not enough: ‘I*t’s important to be able to look after yourself’.* The overarching aim should be to give **all** CAMHS leavers, including those discharged to primary care, the confidence, knowledge and support to take responsibility for their own health care and, hence, to flourish in an adult world, by alleviating anxiety, fear and uncertainty.

### Recommendations regarding CAMHS transition procedures

#### Transition age

Young people approved of the youth service model up to the age of 25. However, to our knowledge such major transformation was not under consideration by the transition review panels in the two trusts so, in the interests of deliverability, young people recommended a flexible transition age of 18/19, but that age should not be the primary or sole criterion for transition, ‘w*e don’t feel like adults, just because we’ve turned 18*’.

#### Time and pace

Young people and parents called for gradual transitions with time for young people to progress to readiness at their own pace to avoid the ‘cliff edge of lost support’ [27]. The six-month planning period recommended in guidance [17] was considered reasonable but should be flexible. Young people need time to *‘… get used to the idea and all the space and facility to ask questions … It is a frightening experience for some not to have the security of possibly the one person they have built a trusting relationship with.*’ [parent]. Many clinicians felt hampered by lack of time (and lack of resources), ‘*we often have to just do what is quickest and cheapest*’. *Y*oung people and parents called for ongoing support when young people leave CAMHS, ‘*… not a quick closure … but a time of transition e.g. groups for certain people or a length of time when the young person still has contact with a therapist or a phone service’* [parent].

#### Flexibility

Young people called for flexible transitions which account for individual need, life context and readiness with inbuilt second chances when mistakes are made. Transition should not occur during a mental health or personal crisis.

Many clinicians agreed that flexibility would be beneficial and suggested a ‘*transition window … eg between age 16–25, but mostly driven by young person’s subjective wellbeing*’. However, many thought such flexibility would be difficult to achieve as ‘*services don't like vague cut offs, they like clarity’*. Clinicians identified a group of reluctant CAMHS leavers who ‘… *might not choose to be adults’*. Some questioned the therapeutic value to these young people, of delaying the move to adult services.

The workshops identified an important disconnect: young people said they are least able to cope with the move when they are most unwell as they are unable to make decisions, organise themselves or concentrate. They want be able to move when they are relatively well and most able to cope. However, high AMHS thresholds dictate that only the most unwell will be accepted ‘*if you are well you won't qualify for a service’* [clinician].

#### Shared decision-making (SDM)

Young people wanted to be involved in decisions about their health care, including at transition. Time and opportunities to discuss and reflect are essential to the decision-making process. They warned against tokenistic commitment to SDM. Young people agreed that the decision about the role of parents at transition should sit firmly with the young person.

#### Asset-focused

Young people and parents agreed that preparation should be asset- rather than deficit-focused. Leavers should be supported to identify and develop a range of personal skills and assets to empower them at transition and into adulthood. Building self-confidence was seen as key, along with resilience, help-seeking, coping strategies, self-esteem, organisation and social skills.

#### GOAL directed

Transition preparation should be goal directed with progress carefully monitored and recorded. Young people considered various models.

### Tools for delivering the TPP

The consensus among young people was that take up of preparation activities would vary: some CAMHS leavers would embrace many of the preparation activities on offer, where others may require few. This should be accepted by services. Imposing the TPP would defeat the object of a personalised programme in which young people feel they have, at least some, control.

#### Red flags

A red-flag system should alert clinicians six months before a young person reaches transition age to trigger preparation, at which point the clinician would outline the range of preparation activities on offer.

#### Joint working

Joint meetings between the young person, the referring and the accepting clinicians (and parent/carer depending on the young person’s wishes) were called for along with improved channels of communication. Clinicians agreed that a ‘*cross-over period*’ would improve continuity of care. Parents recommended that *‘adult and young people’s services* [should] *work together to see what steps need to be taken to help the young person in the adult phase of their life’* [parent]. AMHS clinicians were keen to avoid inappropriate referrals to AMHS which are disappointing for young people and time-consuming and expensive for services. They suggested routine pre-referral CAMHS/AMHS discussions **before** the possibility of ongoing referral is mentioned to a young person.

#### Transition peer support workers

Dedicated transition peer support workers (TPSW) with CAMHS experience should guide and support young people through their preparation period, individually and in small groups, to identify and reach their transition goals by: accompanying a young person to their first AMHS appointment; organising visits to AMHS and other third sector organisations; answering questions and fact-finding; advocacy and liaison; sourcing, maintaining and distributing information; organising and facilitating small group activities some of which may include visiting speakers to advise on specific, relevant topics such as legal rights, benefits or housing, according to individual needs and interests.

The roles should be paid, well supervised and offer accredited training. Clinicians agreed that good support, training and supervision would be essential but voiced concerns about the capacity of the service to provide this. Clinicians identified other potential difficulties as funding, recruitment difficulties, attitudes of professionals, confidentiality, concerns about the wellbeing of the TPSW and their ability to accept responsibility. Two clinicians were not in favour of TPSWs because ‘*introducing another person might not help*’.

Young people saw the TPSW as the mainstay of the programme and felt all CAMHS leavers should be introduced to a TPSW at the beginning of the preparation period with continued, open and easy access should they find themselves in need of support or information.

#### Transition booklet

Young people in Tr1 designed a prototype booklet, accompanied by user guidelines, to present to service providers and commissioners. The small A5 folder, divided into easy-to-use sections enables the user to build up a useful, personalised resource. This should be introduced by the TPSW but completed and held by the young person. Young people were against preparation by questionnaire. Most were familiar with, but bored by, questionnaires, viewing them as a convenience for practitioners rather than an engaging way of meeting the needs of young service users. The transition booklet is the antithesis of a questionnaire approach.

The transition booklets would provide focus and structure to the preparation process. Working with the TPSW (and clinician or other nominated person), a young person would compile their booklet during the preparation period according to their personal needs, wishes and interests, identifying goals, barriers, areas of need, recording progress, achievements, milestones and decisions. Young people would take responsibility for their booklet, deciding what to share, who with and when.

An information section would include case studies, frequently asked questions, resource lists (including crisis), contact details, maps, appointment procedures, service procedures and therapies.

A personal section should cover: biographical details (including interests, hobbies, likes/dislikes to remind clinicians of individuality); the identification of personal assets likely to help now and in the future, including resilience, positive coping strategies, self-esteem and confidence; understanding and looking after myself (including mental and physical health); when and how to get help; parental involvement; life context (financial situation, family problems, upcoming exams, relationships problems); identifying supporters who are ‘*on my side’* and to offer support across transition.

#### Information

Good quality, relevant, accessible and up-to-date information, co-produced by young people, was seen as key to ease anxiety and uncertainty: *‘the right information, from the right people, at the right time’*. Information shouldn’t be ‘*thrown at people’,* but presented gradually, with time to reflect, question and discuss and be available in a variety of formats (apps, online, leaflets, flyers, posters, visits and virtual clinic tours). Clinicians voiced concern about the funding and staff to maintain these resources and identified their own information requirements, particularly about third sector organisations about which they often know very little and cannot guarantee quality. Parents requested information on how to support their children across the transition.

### Existing policy and guidance

At the onset of the study young people were unaware of the national or local transition policy, guidance or protocols. Key points from these were introduced and discussed at stage 3. Participants’ recommendations closely accorded with policies and current guidance [15, 17]. For example, a logbook was suggested by Singh and colleagues in 2010 [7] and, during stage 3 workshops, young people viewed the recently-launched NHS mental health passport [28]. The NHS template was considered too formal and participants decided to design a bespoke version, attractive and engaging for young people.

### Evaluation

In brief, young people found the creative methods conducive to ‘*thinking outside the box’* and valued the mix of activities which changed the pace of the day, maintained energy levels and interest ‘*because it’s fun we are more interested in giving ideas instead of just sitting and talking*’. Young people felt at ease and able to talk openly in ‘*a positive and interactive environment’* [PC]. PCs agreed that young people were relaxed and stimulated which aided concentration and facilitated discussions. Although the workshops were a considerable commitment for young people, they provided valued time and space to explore, share and think. Young people felt listened to ‘*you guys, like, listen. Didn’t feel like research’* [23].

## Discussion

The study aimed to co-design, with CAMHS users, recent leavers and clinical staff, a CAMHS Transition Preparation Programme (TPP) deliverable in routine NHS mental health settings. Our creative, participatory approaches enabled participants to give serious consideration to the issues and generate original ideas and practical recommendations.

The young people’s recommendations, aims and suggested tools for delivery were informed by their largely negative perceptions and experiences of mental health services and CAMHS transition. Most had felt uninvolved, unprepared and poorly informed. As a result, they had felt anxious, uncertain and fearful. They viewed CAMHS transition as one of the multitude of changes and demands in their lives and wished services to do the same. They were clear that teenagers make mistakes and ‘*don’t just grow up overnight*’. They called for services to work together to provide time, flexibility and non-punitive procedures.

Their sense of estrangement from support services may be particularly detrimental at a time when they are required to take responsibility for their own health care whilst grappling with the ongoing demands of adolescence. It seems unsurprising that young people’s service engagement significantly declines at this age [29] but highlights the urgent requirement to design mental health services which support young people to embark upon independence with confidence, not fear. Transition preparation should aim to enable young people to leave CAMHS with the confidence, knowledge and the personal strengths to fit them for adult life, not just life in adult services. Young people want services to fit the needs of the client group, not the reverse.

On the whole, clinical staff viewed favourably the young people’s recommendations. Practitioners from both child/adolescent and adult services acknowledged the need for improved communication and understanding between all relevant support services, both NHS and third sector, to improve transitions for young people. Culture change and commissioner buy-in were seen as essential to facilitate meaningful change.

The study aimed to move beyond comment and policy review, to offer practical solutions to mental health service providers. The young people were keen to take a pragmatic approach to improve service delivery at transition. Our close NHS collaboration ensured the study had a high profile in participating trusts. CAMHS transition reviews were underway in two participating trusts. The author, young people and PCs were asked to feed study findings into these review panels to inform protocol and service redesign. Similarly, we cascaded findings within the trusts by presenting to trust boards, senior management and team meetings. These two Trusts are committed to implementation: ‘*the project has already and will continue to have a big impact … A transitions working group has been set up which is looking to use and build on the research as the foundation for changing and improving practice, processes, policy and performance … Some of the young people who participated in the research are now part of this group … to see their ideas … come to fruition in practice.’* (chair, transition working group, Tr2).

Studies concerning the effectiveness of interventions are rare and many focus on restructured youth services [4, 30] which were not the focus of this study. Many of the themes to emerge from this study aligned with previous research and guidance: the importance of a flexible, gradual, holistic process, individualised transition plans, improved joint working, continuing support and communication between services [3–7, 15–17]. One example, the Ready Steady Go programme [9] is a generic transition programme employed in routine care across services in a large UK NHS hospital. In line with the TPP, it aims to empower young people at transition. To our knowledge, young people were not integral to the design of these tools. Ready Steady Go and other examples of transition readiness assessments for patients with chronic illness [8–11] use batteries of tick-box questionnaires to highlight needs and assess readiness. The young people in this study were not in favour of preparation through questionnaire, opting instead to steer themselves through a programme of activities, coordinated and supported by peer support workers.

The young people’s TPP described here, differs from guidance and policy [14, 15, 17, 18] in its expanded definition of transition, to include **all** CAMHS leavers, including those discharged to primary care. The young people felt services underestimated the fear, anxiety and uncertainty young people experience when they leave CAMHS, irrespective of discharge or transfer. Preparation, they said, should be offered to all young people facing these major changes in their care arrangements.

## Limitations

Our sample size is small and participants were recruited from pre-existing participation networks which may not represent the wider CAMHS population. Specific groups may have particular requirements not covered here.

Creative, participative methodologies have been criticised as lacking rigour, quality control and being ‘at risk for an “anything goes” criteria’ [31]. These issues were addressed by bringing together a team of experienced professionals from NHS, research and youth work who agreed and shared clearly stated aims, through our process of ongoing consensus and through the independent evaluation [23].

Although there is no standard optimal group size for creative workshops, very small numbers may restrict the range of activities deliverable, narrow the range of experiences in the pool and enable strong personalities to dominate. However, our workshop facilitator was alert to group dynamics and the need to be inclusive. Our evaluation showed that many young people preferred these small groups. Governance procedures were more time-consuming than anticipated which delayed recruitment, resulted in a small sample and insufficient time to organise and train young people for some of the planned co-research activities.

## Conclusion

A CAMHS Transition Preparation Programme (TPP) designed by young people for young people, with input from clinical staff, should be engaging, relevant and deliverable in routine mental health settings. Young people want the transition process to help **all** CAMHS leavers build confidence, identify sources of support and improve knowledge to enable them to take responsibility for their own care. The innovative methods used in this study were popular with young people and enabled them to think creatively about transition preparation. Robust collaborative working, including multi-disciplinary research teams, resulted in the study findings informing CAMHS transition reviews in two trusts, committed to implementation.

## Abbreviations

AMHS: Adult mental health services
CAMHS: Child and adolescent mental health services
CLAHRC: Collaborations for Leadership in Health Research and Care
CPFT: Cambridgeshire and Peterborough NHS Foundation Trust
FB: Feedback
HPFT: Hertfordshire Partnership University NHS Foundation Trust
LR: Lead researcher
NEET: Not in education, employment or training
NHS: National Health Services
NIHR: National Institute for Health Research
NSFT: Norfolk and Suffolk NHS Foundation Trust
PC: Participation coordinator
SD: Scaffolded discussion
TPP: Transition preparation programme
Tr1,2,3: Trust 1, trust 2, trust 3
